# Investigation of 6-thioguanine as a strategy to overcome methotrexate resistance in a mouse model of leptomeningeal carcinomatosis

**DOI:** 10.1007/s11060-025-05321-5

**Published:** 2025-11-03

**Authors:** Hidemitsu Nakagawa, Yoshihiro Yui, Tsuyoshi Suzuki, Masakazu Tamura, Masanobu Yamada, Masashi Kawaichi

**Affiliations:** 1 Department of Neurosurgery, Nozaki Tokushukai Hospital, 10-50 Tanigawa, 2-Chome, Daito City, Osaka 574-0074 Japan; 2 Nihon Seimei Hospital, Osaka City, Osaka Japan; 3 Research Institute, Nozaki Tokushukai Hospital, Daito City, Osaka Japan

**Keywords:** Leptomeningeal carcinomatosis, MTX resistance, 6-TG, Sequential therapy

## Abstract

**Purpose:**

Leptomeningeal carcinomatosis (LC), the dissemination of malignant cells into the cerebrospinal fluid, occurs in 3–5% of patients with solid tumors and is being recognized more frequently due to prolonged survival with systemic therapies. The prognosis remains dismal, with a median survival of 4–8 weeks. Methotrexate (MTX), the current standard treatment, is often compromised by resistance through dihydrofolate reductase (DHFR) upregulation and by neurotoxicity at high doses, underscoring the need for alternative therapeutic approaches.

**Methods:**

An MTX-resistant subline (R-MM46) was established from a murine mammary carcinoma. Resistance was confirmed by increased DHFR activity, enhanced drug efflux, and apoptosis resistance, and validated in an LC mouse model. Metabolic alterations were assessed by measuring phosphoribosyl pyrophosphate (PRPP), hypoxanthine-guanine phosphoribosyltransferase (HGPRT), and thymidine kinase (TK). The therapeutic efficacy of 6-thioguanine (6-TG), which targets the salvage pathway, was evaluated in vivo.

**Results:**

R-MM46 cells exhibited a 6–7-fold increase in DHFR activity, together with upregulation of P-glycoprotein and Bcl-2. In the LC mouse model inoculated with R-MM46 cells, MTX treatment failed to prolong survival. R-MM46 cells demonstrated PRPP accumulation and increased HGPRT and TK activity, consistent with activation of the salvage pathway. Oral 6-TG significantly extended survival, with the greatest benefit observed when administered sequentially 2–6 h after MTX.

**Conclusion:**

Sequential 6-TG administration capitalizes on salvage pathway activation in MTX-resistant LC and may represent a promising therapeutic strategy to overcome MTX resistance.

**Supplementary Information:**

The online version contains supplementary material available at 10.1007/s11060-025-05321-5.

## Introduction

Leptomeningeal carcinomatosis (LC), defined as the dissemination of malignant cells into the cerebrospinal fluid (CSF), occurs in approximately 3–5% of patients with solid tumors. With advances in systemic chemotherapy and molecular targeted therapies extending survival in primary tumors, LC is increasingly recognized at advanced stages. Currently, intrathecal chemotherapy with methotrexate (MTX), cytosine arabinoside (Ara-C), or thiotepa is considered the standard of care [1–5]⁠. Additional approaches, including molecular targeted agents, immune checkpoint inhibitors, and radiotherapy, have also been explored [6–11]⁠. Nevertheless, prognosis remains poor, with a median survival of only 4–8 weeks from diagnosis, and even with treatment, survival rarely exceeds several months. Resistance to MTX or Ara-C is considered a major barrier to therapeutic efficacy [12–14]⁠; in this study, we focused on MTX resistance as a critical obstacle to improved outcomes.

Multiple mechanisms contribute to MTX resistance, including upregulation of dihydrofolate reductase (DHFR), reduced drug uptake due to impaired transporters, abnormalities in folate polyglutamylation, enhanced salvage pathway activity, and increased drug efflux [15–18]⁠. Among these, DHFR overexpression is regarded as the predominant mechanism [16, 19, 20]⁠. Theoretically, overcoming resistance requires MTX doses exceeding DHFR capacity; however, high-dose MTX is associated with severe neurotoxicity, rendering this approach clinically unfeasible. Thus, novel therapeutic strategies are urgently needed.

Nucleotide metabolism involves both *de novo* synthesis and salvage pathways. In purine metabolism, phosphoribosyl pyrophosphate (PRPP) serves as a ribose donor for *de novo* synthesis of AMP and GMP via IMP. In the salvage pathway, hypoxanthine-guanine phosphoribosyltransferase (HGPRT) reutilizes hypoxanthine and guanine by conjugating them with PRPP to regenerate IMP and GMP. Similarly, nucleoside salvage enzymes such as thymidine kinase (TK) phosphorylate nucleosides to nucleotides, thereby replenishing the pool required for DNA synthesis. These pathways act complementarily; when *de novo* synthesis is inhibited, PRPP accumulates and drives compensatory salvage pathway activation. MTX, as an antifolate, inhibits DHFR and thereby blocks *de novo* purine and thymidylate synthesis. Consequently, PRPP accumulates, enhancing salvage pathway activity and contributing to drug resistance [21–24]⁠.

6-Thioguanine (6-TG), a purine analogue, is incorporated into cells via the salvage pathway. Specifically, HGPRT catalyzes its conjugation with PRPP to form thioguanine nucleotides (TGNs), which are incorporated into DNA, disrupting replication and inducing apoptosis. Under conditions where MTX blocks *de novo* synthesis and PRPP accumulates, 6-TG uptake is enhanced, resulting in potent antitumor activity [25]⁠. Compared with 6-mercaptopurine (6-MP), 6-TG is more efficiently converted into active TGNs, requires fewer metabolic steps, and exhibits stronger cytotoxicity at lower concentrations [26–28]⁠. Previous studies have demonstrated that intracellular TGN levels are higher with 6-TG than with 6-MP, particularly in hematopoietic cells. Moreover, unlike other intrathecal agents, 6-TG can be administered orally, providing advantages in terms of patient convenience and ease of treatment delivery [29–31]⁠.

Based on this rationale, we established an LC mouse model using MTX-resistant tumor cells, characterized its pathological features, and investigated strategies to overcome resistance. In particular, we focused on alterations in nucleotide metabolism, especially salvage pathway activation in resistant cells, and evaluated the therapeutic efficacy of 6-TG in exploiting this metabolic vulnerability.

## Materials and methods

### Cell culture and establishment of MTX-resistant subline

**Fig. 1:**
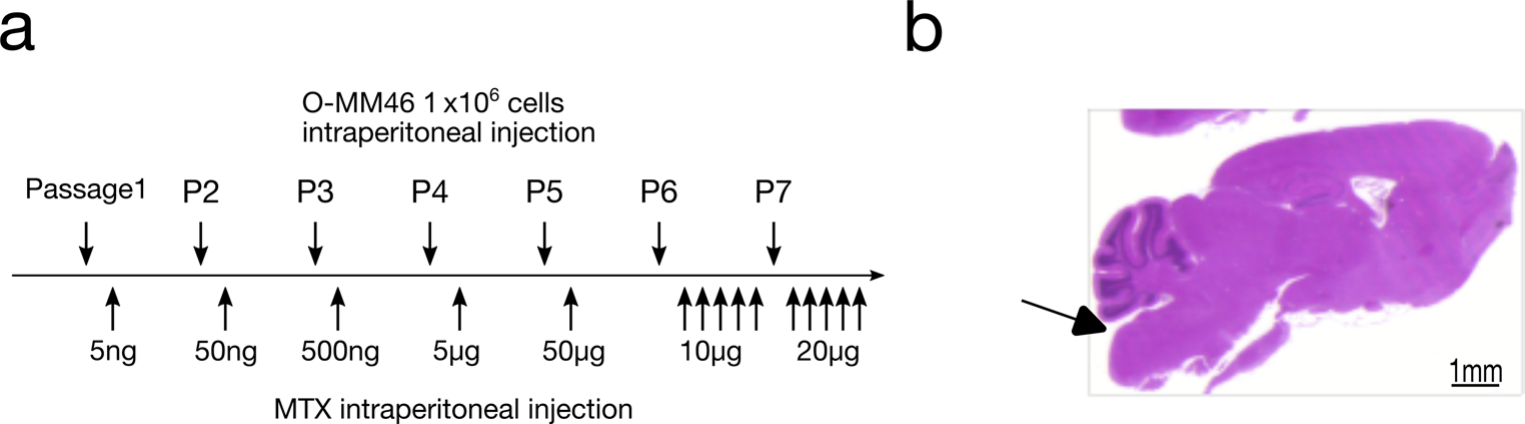
Establishment of experimental models. **a** Procedure for establishing the MTX-resistant R-MM46 cell line. **b** Generation of the leptomeningeal carcinomatosis (LC) mouse model. Tumor cells were injected at the site indicated by the arrow. Scale bar: 1 mm

The mouse mammary carcinoma cell line MM46 (TKG 0163; RRID: CVCL_B429), originally derived from ascites tumors in C3H/He mice, was obtained from the Cell Resource Center for Biomedical Research, Tohoku University (Sendai, Japan). Because MM46 cells are difficult to maintain in vitro, cells (1 × 10⁶) were serially transplanted intraperitoneally into C3H/HeSlc mice (Japan SLC, Shizuoka, Japan) every 12 days. To generate an MTX-resistant subline (R-MM46), MTX was administered intraperitoneally in a stepwise manner at each passage (5 ng to 20 µg), resulting in a stable resistant line (Fig. 1a). The parental line was designated O-MM46. Acquisition of resistance was confirmed by assays for dihydrofolate reductase (DHFR) activity, drug efflux, and apoptosis (see Supplementary Methods) [32–34]⁠.

### LC mouse model

Ten-week-old female C3H/HeSlc mice were used. An LC mouse model with O-MM46 or R-MM46 cells was established by puncturing the cisterna magna under anesthesia with a 26-gauge needle inserted 2 mm (Fig. 1b, arrow), followed by injection of 0.05 ml of cell suspension containing 2 × 10⁵ cells. Mice were maintained under specific pathogen-free conditions with controlled temperature and humidity, a 12 h light/dark cycle, and free access to standard chow and sterilized tap water. Mice were examined daily for body weight, mobility, and food intake. Moribund animals were euthanized and autopsied. Survival time was defined as the interval from tumor cell inoculation to euthanasia. Brains were fixed in 10% formalin, embedded in paraffin, sectioned at 8 μm, and stained with hematoxylin and eosin (H&E). Images were acquired using a phase contrast microscope (Eclipse TE2000-U; Nikon Instruments, Melville, NY) equipped with Plan Fluor 4× (NA 0.13) and 10× (NA 0.30) objective lenses (Nikon Corp, Tokyo, Japan).

### Drug administration

MTX was administered intrathecally. Based on preliminary titration experiments, a dose of 0.25 mg was selected for subsequent studies. 6-TG was administered orally by gavage once daily for five consecutive days. A dose of 0.04 mg was chosen, as it was the highest tolerated without body weight loss in pilot studies. Body weight was monitored daily during and after treatment, and no significant loss was observed in non–tumor-bearing mice.

### Immunostaining

Drug efflux mechanisms were assessed by immunostaining for P-glycoprotein (P-gp), and apoptosis resistance was assessed by staining for Bcl-2. O-MM46 and R-MM46 cells were seeded onto poly-L-lysine–coated slides, fixed, permeabilized, and incubated with the respective antibodies. Detection was performed using DAB, and nuclei were counterstained with hematoxylin. Images were acquired using a phase contrast microscope (Eclipse TE2000-U; Nikon Instruments, Tokyo, Japan) equipped with a Plan Fluor 4× objective lens (NA 0.13; Nikon Corp). Details of antibodies and procedures are provided in the Supplementary Methods.

### Biochemical assays

DHFR activity, PRPP content, and HGPRT and TK activities were measured using established spectrophotometric or radiochemical assays. Full protocols are provided in the Supplementary Methods.

### Statistical analysis

All analyses were performed using R software (version 4.5.0; RRID: SCR_001905). Comparisons between two groups were made with a two-tailed unpaired Student’s t-test. Survival was analyzed by the Kaplan–Meier method and compared using the log-rank test. For multiple groups, overall comparisons were followed by pairwise log-rank tests with Holm adjustment. The false discovery rate was controlled using the Benjamini–Hochberg method. Statistical significance was defined as *P* < 0.05. Data are presented as mean ± SD unless otherwise specified. Plots and survival curves were generated using the ggplot2 (RRID: SCR_014601) and survminer (RRID: SCR_021094) packages.

## Results

### Confirmation of MTX resistance in R-MM46 cells

**Fig. 2:**
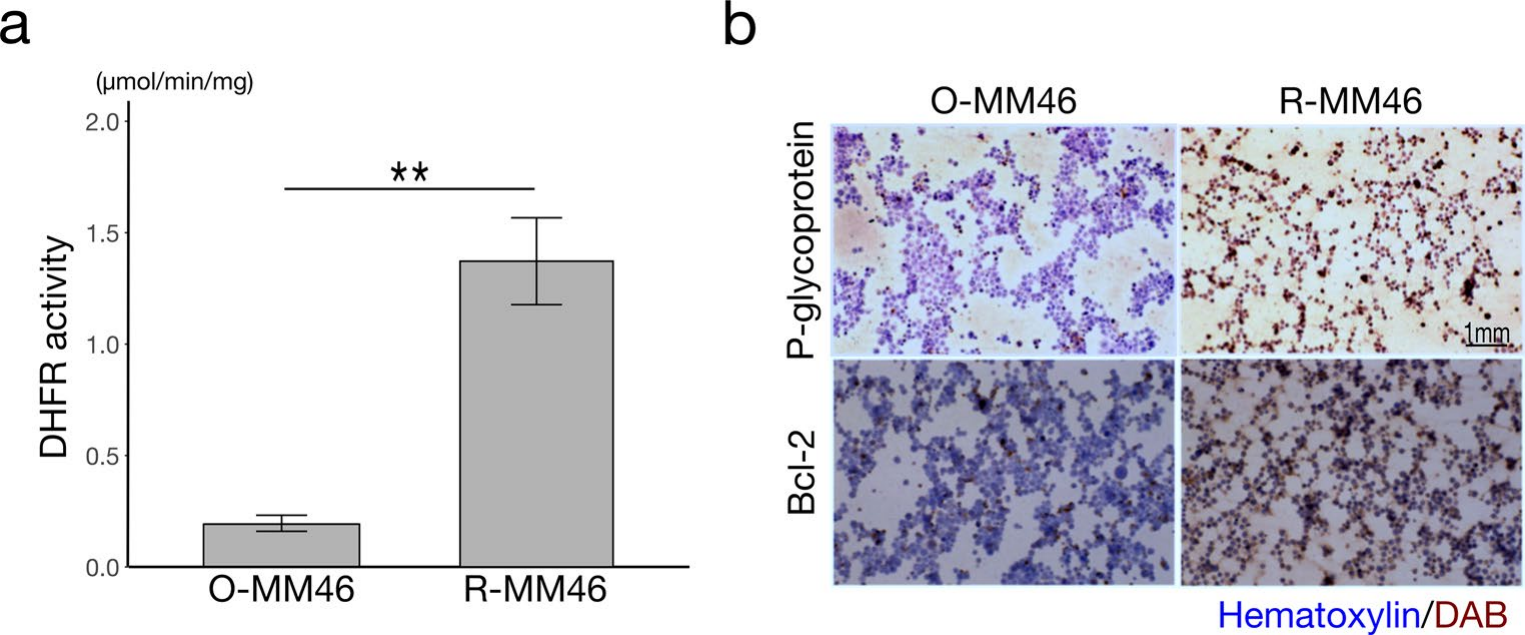
Characterization of MTX resistance in vitro. **a** DHFR activity. Values are mean ± SD (*n* = 5); **p < 0.01. **b** Immunohistochemical staining for P-gp and Bcl-2. Scale bar: 1 mm

The MTX-resistant subline R-MM46 exhibited a 6–7-fold increase in DHFR activity compared with the parental O-MM46 cells (Fig. 2a). Immunostaining for P-gp and Bcl-2 further demonstrated markedly higher positivity in R-MM46 cells (Fig. 2b). These findings confirm that R-MM46 cells acquired enhanced drug efflux capacity, associated with Mdr-1 induction, together with resistance to apoptosis.

### Establishment of the LC mouse model

**Fig. 3:**
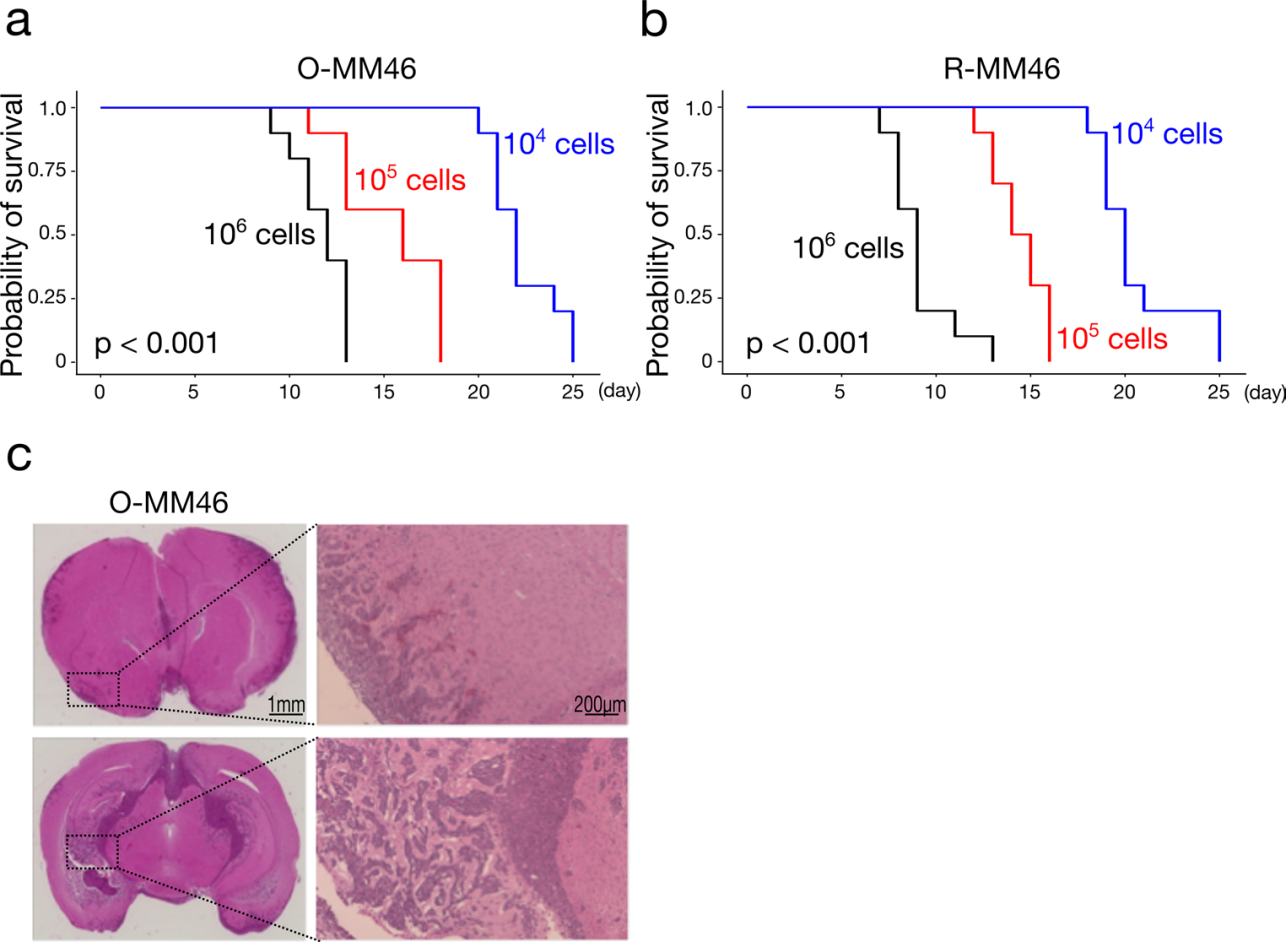
Establishment of the LC mouse model. **a** Survival in mice inoculated with O-MM46 cells. Mean survival (days, mean ± SD): 1 × 10⁶, 11.7 ± 1.42; 1 × 10⁵, 15.4 ± 2.67; 1 × 10⁴, 22.3 ± 1.77. Overall log-rank, *p* < 0.001. Pairwise log-rank (BH-adjusted): 10⁶ vs. 10⁵, *p* = 0.002; 10⁶ vs. 10⁴, *p* < 0.001; 10⁵ vs. 10⁴, *p* < 0.001. **b** Survival in mice inoculated with R-MM46 cells. Mean survival (days, mean ± SD): 1 × 10⁶, 9.3 ± 1.72; 1 × 10⁵, 14.4 ± 1.43; 1 × 10⁴, 20.6 ± 2.46. Overall log-rank, *p* < 0.001. Pairwise log-rank (BH-adjusted): all comparisons, *p* < 0.001. **c** Histology of brain tissues from LC models. O-MM46 cells infiltrating from the subarachnoid space and ventricular cavity into the brain parenchyma. Scale bars: 1 mm (low magnification), 200 μm (high magnification)

To evaluate the in vivo behavior of MTX-resistant cells, LC mouse models were generated with either O-MM46 or R-MM46 cells. In both models, survival time was significantly inversely correlated with the number of injected cells (Fig. 3a, b). At equivalent inoculum doses, mice receiving R-MM46 cells tended to show shorter survival than those inoculated with O-MM46 cells; at 1 × 10⁶ cells, the difference was statistically significant (*p* = 0.009). Histopathological examination revealed tumor cell infiltration from the brain surface into the parenchyma (Fig. 3c), indicating that these models faithfully recapitulate the pathological features of clinical LC.

### Validation of MTX resistance in vivo

**Fig. 4:**
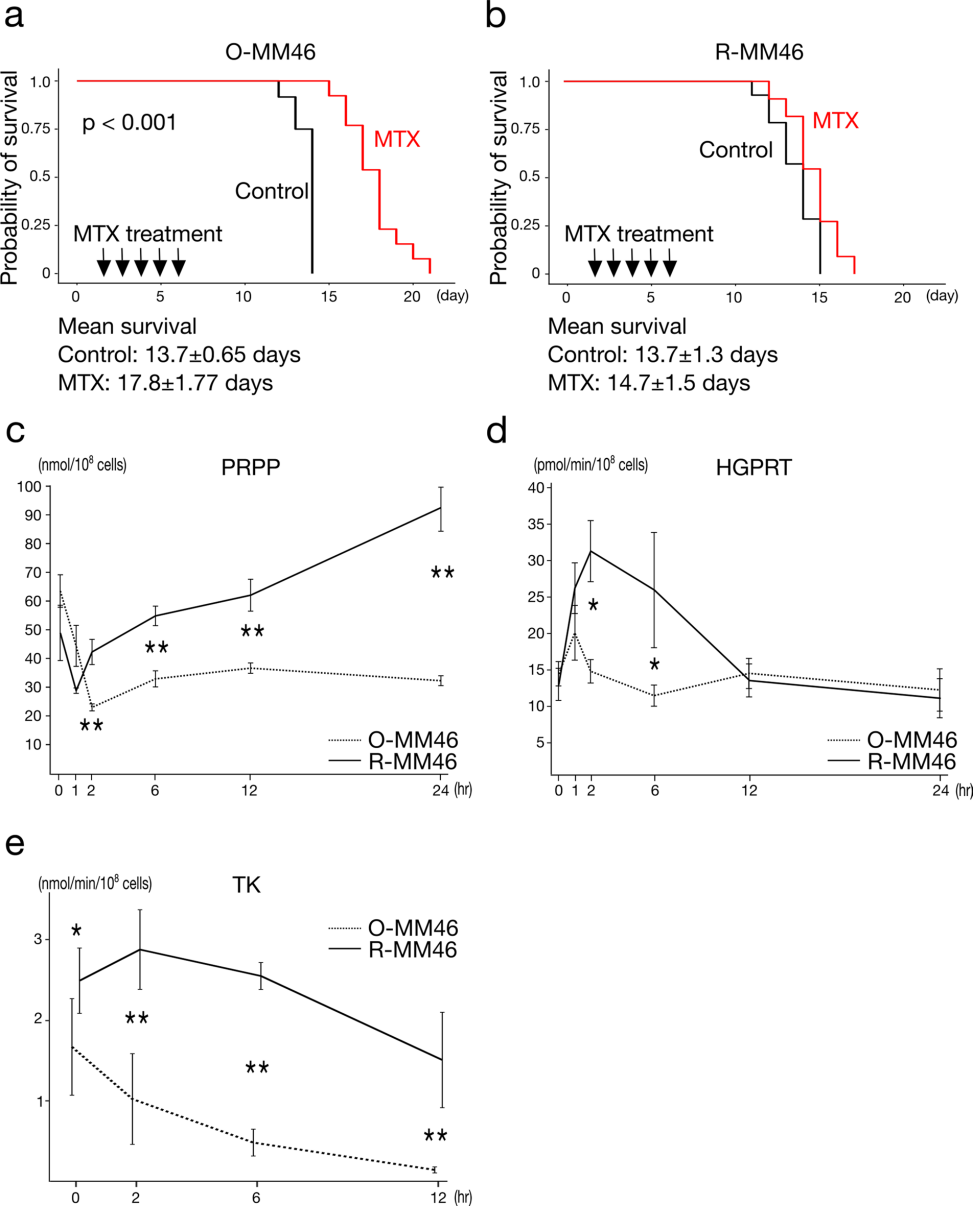
MTX resistance in vivo and associated metabolic adaptations. **a** In the O-MM46 LC model, MTX significantly prolonged survival. Mean survival (days, mean ± SD): Control, 13.7 ± 0.65; MTX, 17.8 ± 1.77. Log-rank: *p* < 0.001. **b** In the R-MM46 LC model, MTX did not prolong survival. Mean survival (days, mean ± SD): Control, 13.7 ± 1.3; MTX, 14.7 ± 1.5. (C–E) Metabolic changes after intraperitoneal MTX (50 mg/kg). **c** PRPP levels; **d** HGPRT activity at baseline and 1, 2, 6, 12, 24 h post-treatment; e TK activity at baseline and 1, 2, 6, 12 h. Values are mean ± SD (*n* = 5); *p < 0.05, **p < 0.01

The resistance phenotype of R-MM46 cells was confirmed in the LC model. MTX significantly prolonged survival in mice inoculated with O-MM46 cells (Fig. 4a). In contrast, MTX had no survival benefit in mice inoculated with R-MM46 cells (Fig. 4b), confirming that R-MM46 cells maintained MTX resistance in vivo.

### Metabolic adaptation associated with MTX resistance

To investigate metabolic alterations linked to MTX resistance, we analyzed nucleotide metabolism with a focus on the salvage pathway. In O-MM46 cells, PRPP levels decreased to 36% of baseline at 2 h post-MTX and remained at ~ 50% thereafter. In contrast, R-MM46 cells showed a transient decrease to 60% at 1 h, followed by progressive accumulation, reaching ~ 130% at 12 h and nearly twice baseline at 24 h (2 h, 6 h, 12 h, 24 h: all *p* < 0.01) (Fig. 4c). HGPRT activity increased in both O-MM46 and R-MM46 cells at 1 h after MTX (140% and 201% of baseline, respectively). In R-MM46 cells, HGPRT activity peaked at 2 h, then declined to levels comparable to O-MM46 by 12 h. HGPRT activity was significantly higher in R-MM46 than O-MM46 at 2 h and 6 h (*p* < 0.05 for both) (Fig. 4d). TK activity in R-MM46 cells was consistently elevated relative to O-MM46, both at baseline and at 2, 6, and 12 h after MTX (baseline: *p* < 0.05; 2 h, 6 h, 12 h: all *p* < 0.01) (Fig. 4e). These results indicate that MTX inhibits *de novo* nucleotide synthesis in R-MM46 cells, leading to impaired PRPP utilization and subsequent PRPP accumulation. This accumulation promotes salvage pathway activation, further augmented by increased HGPRT activity, thereby creating a context favorable for agents such as 6-TG that exploit the salvage pathway. Based on these findings, we hypothesized that MTX combined with 6-TG could overcome resistance and evaluated this strategy in vivo.

### Efficacy of combined treatment with 6-TG

**Fig. 5:**
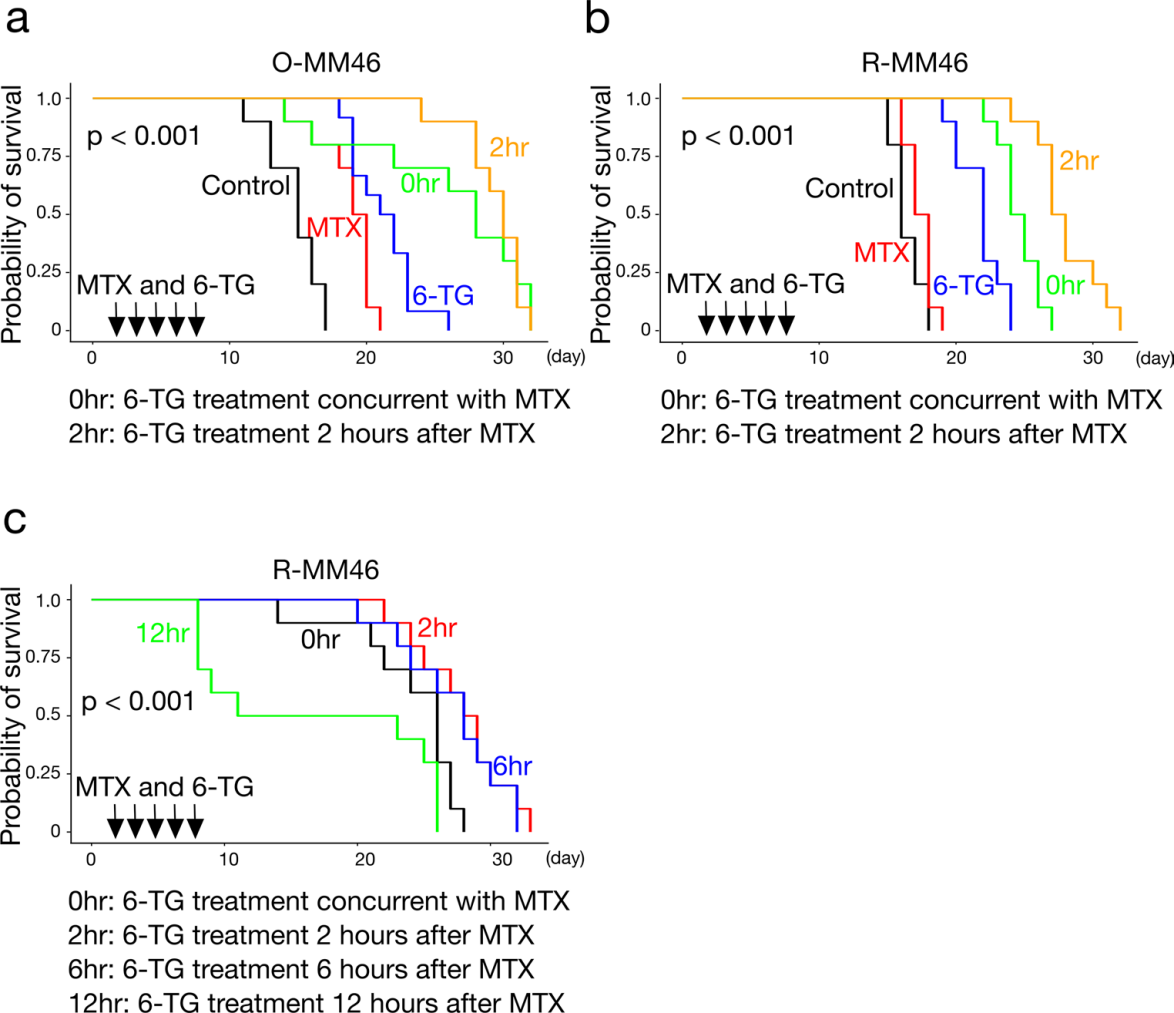
Therapeutic efficacy of MTX and 6-TG. **a** O-MM46 LC model. All treatment groups showed prolonged survival compared with controls. Both MTX and 6-TG monotherapy significantly extended survival. Concurrent MTX + 6-TG (0 h) and sequential administration of 6-TG 2 h after MTX further prolonged survival compared with MTX alone. No significant difference was observed between concurrent and sequential (2 h) administration. Mean survival (days, mean ± SD): Control, 14.8 ± 1.93; MTX, 18.7 ± 2.16; 6-TG, 21.3 ± 2.34; 0 h, 25.9 ± 6.51; 2 h, 29.4 ± 2.32. Overall log-rank: *p* < 0.001. Pairwise log-rank (BH-adjusted): Control vs. MTX, *p* < 0.001; Control vs. 6-TG, *p* < 0.001; Control vs. 0 h, *p* < 0.001; Control vs. 2 h, *p* < 0.001; MTX vs. 6-TG, *p* = 0.014; MTX vs. 0 h, *p* = 0.002; MTX vs. 2 h, *p* < 0.001; 6-TG vs. 0 h, *p* = 0.007; 6-TG vs. 2 h, *p* < 0.001; 0 h vs. 2 h, *p* = 0.64. **b** R-MM46 LC model. All treatment groups except MTX monotherapy significantly prolonged survival compared with controls. 6-TG monotherapy significantly extended survival relative to MTX. Concurrent MTX + 6-TG (0 h) and sequential administration 2 h after MTX further prolonged survival compared with both MTX and 6-TG monotherapy. Sequential 2 h treatment was also superior to concurrent administration. Mean survival (days, mean ± SD): Control, 16.4 ± 1.07; MTX, 17.4 ± 0.97; 6-TG, 21.8 ± 1.69; 0 h, 24.6 ± 1.51; 2 h, 28.0 ± 2.40. Overall log-rank: *p* < 0.001. Pairwise log-rank (BH-adjusted): Control vs. MTX, *p* = 0.058; Control vs. 6-TG, *p* < 0.001; Control vs. 0 h, *p* < 0.001; Control vs. 2 h, *p* < 0.001; MTX vs. 6-TG, *p* = 0.014; MTX vs. 0 h, *p* = 0.002; MTX vs. 2 h, *p* < 0.001; 6-TG vs. 0 h, *p* = 0.001; 6-TG vs. 2 h, *p* < 0.001; 0 h vs. 2 h, *p* < 0.001. **c** Timing of sequential therapy in the R-MM46 LC model. Only sequential administration of 6-TG 2 h after MTX significantly prolonged survival compared with concurrent administration. Administration at 6 h showed a trend toward longer survival, while administration at 12 h resulted in shorter survival. Mean survival (days, mean ± SD): 0 h, 24.1 ± 4.20; 2 h, 27.9 ± 3.48; 6 h, 27.2 ± 3.94; 12 h, 17.0 ± 8.73. Overall log-rank: *p* < 0.001. Pairwise log-rank (BH-adjusted): 0 h vs. 2 h, *p* = 0.027; 0 h vs. 6 h, *p* = 0.050; 0 h vs. 12 h, *p* = 0.057; 2 h vs. 6 h, *p* = 0.672; 2 h vs. 12 h, *p* = 0.011; 6 h vs. 12 h, *p* = 0.013

To test this hypothesis, survival was compared among groups receiving MTX alone, 6-TG alone, or MTX plus 6-TG, administered either concurrently or sequentially. In the O-MM46 LC model, both MTX (*p* < 0.001) and 6-TG monotherapy (*p* < 0.001) significantly prolonged survival compared with controls. Concurrent MTX plus 6-TG (*p* < 0.01) and sequential administration of 6-TG 2 h after MTX (*p* < 0.001) further extended survival compared with MTX monotherapy. However, survival did not differ significantly between sequential and concurrent treatment (Fig. 5a). In the R-MM46 LC model, all treatment groups except MTX monotherapy significantly extended survival compared with controls. 6-TG monotherapy was superior to MTX alone (*p* < 0.05). Both concurrent MTX plus 6-TG (*p* = 0.001) and sequential administration 2 h after MTX (*p* < 0.001) further improved survival compared with 6-TG alone. Notably, sequential administration at 2 h yielded significantly longer survival than concurrent treatment (*p* < 0.001) (Fig. 5b). These results demonstrate that “sequential therapy,” in which 6-TG is delivered at the time of MTX-induced salvage pathway activation, is particularly effective.

### Optimization of sequential therapy timing

Given the benefit of 6-TG at 2 h after MTX, we further examined the impact of timing. In the R-MM46 LC model, survival was compared among groups receiving MTX plus 6-TG administered concurrently, or 6-TG given 2, 6, or 12 h after MTX. Sequential administration at 2 h significantly prolonged survival compared with concurrent treatment (*p* < 0.05). Administration at 6 h showed a trend toward longer survival (*p* = 0.050), whereas administration at 12 h resulted in shorter survival compared with concurrent treatment (Fig. 5c).

## Discussion

This study investigated the mechanisms of MTX resistance in breast cancer–derived cells, with particular emphasis on metabolic alterations. In R-MM46 cells, MTX resistance was associated not only with DHFR overexpression but also with increased expression of Mdr-1 and Bcl-2, which may suggest enhanced drug efflux and resistance to apoptosis. Metabolic reprogramming was evident, characterized by PRPP accumulation together with increased HGPRT and TK activities, suggesting activation of the salvage pathway. Targeting this pathway with 6-TG in combination with MTX significantly prolonged survival in the LC mouse model. Moreover, sequential administration of 6-TG 2 h after MTX—timed to coincide with PRPP accumulation and HGPRT upregulation—was more effective than concurrent administration. Collectively, these findings provide the first evidence in an LC mouse model that sequential 6-TG administration after MTX can circumvent resistance via salvage pathway activation, highlighting its potential as a therapeutic strategy for CNS malignancies.

DHFR amplification and increased activity are well established as primary drivers of MTX resistance. However, evidence for compensatory salvage pathway activation remains limited. Our data showed marked PRPP accumulation and a rapid rise in HGPRT and TK activities in R-MM46 cells, whereas O-MM46 cells exhibited a gradual decline. Although these results do not directly demonstrate inhibition of the *de novo* nucleotide synthesis pathway by MTX, they indicate metabolic reprogramming toward enhanced utilization of the salvage pathway under these conditions, consistent with the classical notion that elevated PRPP drives reliance on the salvage pathway [21–24]⁠. Under these conditions, 6-TG is efficiently activated by HGPRT and incorporated into DNA, inducing cell death through replication stress and DNA damage. Previous reports have suggested that sequential administration—delivering 6-TG after MTX-induced PRPP accumulation—enhances nucleotide incorporation and cytotoxicity [25]⁠. Consistent with this, our study demonstrated that 6-TG given 2–6 h after MTX prolonged survival more effectively than concurrent treatment. By contrast, administration at 12 h was less effective, likely reflecting attenuation of salvage pathway activation by that time, with additive toxicity predominating [35, 36]⁠.

Previous studies have indicated that upregulation of Mdr-1 and Bcl-2 commonly represents a secondary adaptation to chemotherapeutic stress rather than a primary cause of resistance [37–39]. In line with this concept, the present study suggests that the major mechanism underlying MTX resistance in R-MM46 cells involves a metabolic shift from the de novo to the salvage pathway. Accordingly, the increased expression of Mdr-1 and Bcl-2 observed in R-MM46 cells is likely to reflect a downstream adaptive response to MTX exposure rather than a direct driver of resistance.

Interestingly, in R-MM46 cells, a paradoxical phenomenon was observed in which PRPP accumulated and remained at high levels despite increased DHFR activity and enhanced consumption through HGPRT. This can be explained by the fact that, under MTX exposure, the flux through the *de novo* purine synthesis pathway is not fully maintained, resulting in insufficient PRPP utilization via this route and diversion of excess PRPP into the salvage pathway. In R-MM46 cells, HGPRT activity is rapidly induced, establishing a state in which PRPP can be efficiently utilized through the salvage pathway; however, the amount consumed does not exceed its production and inflow, leading to the simultaneous observation of PRPP accumulation and HGPRT activation. Moreover, the precise reason why R-MM46 cells exhibit greater PRPP accumulation than O-MM46 cells remains unclear, but overproduction of PRPP or reduced utilization by alternative pathways may be involved.

It should also be noted that mice maintained under standard laboratory conditions exhibit substantially higher circulating folate concentrations (approximately 20–45 ng/mL) [40, 41] than those observed in humans (approximately 3–20 ng/mL) [42]. Therefore, the antifolate effect of MTX might be partially attenuated in mice, resulting in stronger activation of resistance mechanisms such as DHFR upregulation and salvage pathway dependence. Consequently, the therapeutic benefit of sequential 6-TG administration observed in this study may appear more pronounced than under physiological human folate levels. To better predict clinical applicability, future studies using low-folate diets or other approaches to mimic physiological folate conditions will be required.

Our results also demonstrated that orally administered 6-TG can penetrate the CNS and exert therapeutic effects. In this study, however, we did not directly confirm whether 6-TG effectively engaged its intended target. Future studies aiming at clinical translation should include quantification of thioguanine nucleotides (TGN) and DNA-incorporated 6-TG bases (e.g., by HPLC or LC-MS/MS) to provide direct biochemical evidence that MTX pretreatment enhances 6-TG activation through PRPP accumulation and HGPRT pathway stimulation. In addition, most prior pharmacokinetic studies examined continuous infusion, and CSF penetration after once-daily oral dosing, as used here, remains insufficiently characterized. Future studies should therefore include quantification of CSF 6-TG concentrations after oral administration and comprehensive safety evaluation, including the risk of neurotoxicity. Additional issues warranting investigation include: (i) validation in other tumor types and patient-derived or PDO models; (ii) exploration of multidrug strategies beyond folate pathway inhibition and salvage pathway targeting; and (iii) identification of predictive biomarkers of efficacy through CSF metabolomic profiling.

Several limitations should be acknowledged. First, the study was limited to a single mouse strain and tumor cell line, restricting extrapolation to other tumor types or clinical cases. Second, the role of the immune system was not addressed, leaving potential interactions with immunosuppressive agents or host immune responses unexplored. Third, systemic toxicities, including the risk of 6-TG–induced myelosuppression, were not comprehensively assessed; clinical application will require strict safety management with dose optimization and therapeutic drug monitoring (TDM). In addition, MM46 cells require in vivo serial passage due to their limited capacity for continuous in vitro culture, raising animal welfare concerns. Nevertheless, MM46 cells have long served as a reliable model for studying MTX resistance mechanisms, justifying their selection in this study.

## Conclusion and future directions

Our LC mouse model demonstrated that timed administration of 6-TG after MTX effectively exploited salvage pathway activation and significantly prolonged survival. These findings suggest that inhibition of the salvage pathway by 6-TG represents a promising strategy to overcome MTX resistance. Future work should prioritize pharmacokinetic characterization, including CSF concentration measurements and neurotoxicity assessment, as well as optimization of sequential dosing schedules, with the ultimate goal of clinical translation.

## Supplementary Information

Below is the link to the electronic supplementary material.

Supplementary Material 1

## Data Availability

The datasets generated during and/or analyzed during the current study are available from the corresponding author on reasonable request.
