# Supplementary material for: Investigation of 6-thioguanine as a strategy to overcome methotrexate resistance in a mouse model of leptomeningeal carcinomatosis
Source: J Neurooncol. 2025 Nov 3;176(1):64. doi: 10.1007/s11060-025-05321-5 (PMC12583346; doi:10.1007/s11060-025-05321-5)
Supplement: Supplementary file 1 — Supplementary Material 1 [file 11060_2025_5321_MOESM1_ESM.pdf]

Supplementary Information

Title

Investigation of 6-Thioguanine as a Strategy to Overcome Methotrexate resistance in a Mouse Model of Leptomeningeal Carcinomatosis

Journal Name

Journal of Neuro-Oncology

Author Names

Hidemitsu Nakagawa, Yoshihiro Yui, Tsuyoshi Suzuki, Masakazu Tamura, Masanobu Yamada, Masashi Kawaichi

*Correspondence

Hidemitsu Nakagawa

Dept. of Neurosurgery, Nozaki Tokushukai Hospital, 10-50 Tanigawa, 2-Chome, Daitou, Osaka 574-0074, Japan

Phone: 81-72-874-1641; Fax: 81-72-818-3723; E-mail: nakagawa. hide@tokushukai.jp

Supplementary methods

Antibodies and reagents

Mouse monoclonal anti-p-glycoprotein antibody (#03-57043; RRID: AB_1542734) was purchased from ARP American Research Products, Inc. (Waltham, MA). Mouse monoclonal anti-Bcl-2 antibody (# M0887; RRID: AB_2064429) was purchased from DAKO/Agilent (Santa Clara, USA). Peroxidase Stain DAB Kit (#25985) was purchased from NACALAI TESQUE, Inc. (Kyoto, Japan). MTX (#1230) for injection was purchased from R&D Systems (Minneapolis, USA). 6-TG (#T0212) was purchased from Tokyo Chemical Industry Co., Ltd. (Tokyo, Japan).

DHFR activity assay

Dihydrofolate reductase (DHFR) activity was measured in ascitic tumor cells from O-MM46 and R-MM46, with five independent samples for each group. All procedures were carried out at 4 °C. Tumor cells were harvested from the peritoneal cavity and centrifuged at 450 × g for 10 min, and the supernatant was discarded. A total of 1×10^8 cells were homogenized in 0.3 ml of buffer (100 mM Tris-HCl, pH 8.0, containing 10 mM 2-mercaptoethanol and 150 mM KCl) and stored at –90 °C. After thawing, the homogenates were centrifuged at 105,000 × g for 20 min, and the resulting supernatant was used for enzymatic assays. DHFR activity was determined spectrophotometrically by monitoring the decrease in absorbance at 340 nm. The molar extinction coefficients of DHF and NADPH at 340 nm were $\varepsilon = 5.1\ mM^{-1}\ cm^{-1}$ and $\varepsilon = 6.2\ mM^{-1}\ cm^{-1}$, respectively. The conversion of 1 mmol of DHF to THF catalyzed by DHFR results in a total absorbance decrease of 11.3 ($\Delta\varepsilon = 5.1$ for DHF→THF, $\Delta\varepsilon = 6.2$ for NADPH→NADP). Enzyme activity was calculated from the rate of absorbance decline. Total protein concentration was determined using the Bradford assay (or alternatively the BCA assay), and DHFR activity was normalized to protein content and expressed as μmol/min/mg protein. This assay procedure was adapted from White et al [1].

Assays of PRPP Content, HGPRT Activity, and TK Activity

PRPP content and activities of HGPRT and TK were measured in O-MM46 and R-MM46 cells. Eight days after intraperitoneal passage, mice received intraperitoneal MTX at 50 mg/kg. Ascitic tumor cells were collected immediately before MTX treatment and at 1, 2, 6, 12, and 24 h thereafter. To remove erythrocytes and leukocytes, freshly harvested cells were mixed 1:1 (v/v) with normal saline and distilled water and kept on crushed ice. The

suspension was centrifuged at 100 × g for 5 min at 4 °C, and the hemolysate supernatant was aspirated. Cells were washed with PBS and centrifuged again at ~100 × g for 5 min at 4 °C, and the remaining red cells were removed. After cell counting, the pellet was centrifuged at ~300 × g for 5 min at 4 °C. All procedures were performed at 4 °C, and cell pellets were stored at –120 °C until assay.

PRPP assay: PRPP content in aliquots of 1×10^8 tumor cells was determined according to the method of Hisata [2]. Briefly, cells were homogenized in ice-cold 0.4 N perchloric acid containing [^{3}H]-PRPP as an internal standard, and the extracts were neutralized with 2 M $KHCO_3$. After charcoal treatment to remove inhibitory nucleotides, PRPP was enzymatically converted to AMP by adenine phosphoribosyltransferase in the presence of [8-^{14}C] adenine. The AMP produced was purified by ion-exchange chromatography, and radioactivity was measured by liquid scintillation counting. PRPP levels were calculated with correction for recovery using the internal standard.

HGPRT assay: Phosphorylation of guanine to GMP catalyzed by HGPRT was assayed in a final reaction volume of 0.25 ml containing 50 mM Tris-HCl (pH 8.0), 50 mM $MgCl_2$, 10 mM NaF, 4 mM PRPP, 10 µM [8-^{3}H]-guanine (148 kBq), 50 µl water, and 150 µl enzyme solution. Incubation was performed at 37 °C for 30 min, and reactions were terminated with 50 µl of 2 M perchloric acid followed by centrifugation at ~600 × g for 10 min. Supernatants (100 µl) were neutralized with 30 µl of 2 M KOH, and 20 µl aliquots were applied to PEI-cellulose thin-layer chromatography using 0.5 M K_2HPO_4 as the mobile phase. GMP spots were scraped, extracted with 0.1 ml of 4 M HCl, and mixed with 10 ml of Scintillation fluid (ACS-II Scintillation Cocktail, cat. no. NACS204; Amersham [now Cytiva], Buckinghamshire, UK). Radioactivity was measured with a liquid scintillation counter. [3, 4]

TK assay: TK activity in tumor cells was determined using the [^{3}H]-thymidine DEAE filter-binding method. Frozen cells were thawed and suspended in extraction buffer (50 mM Tris-HCl, pH 7.5, 10 mM $MgCl_2$, 1 mM DTT, 10 mM NaF, and 0.1% Triton X-100), followed by brief sonication on ice. Lysates were centrifuged at 12,000 × g for 10 min, and the supernatant was used as the enzyme source. Reactions were carried out in a final volume of 50 µl containing 50 mM Tris-HCl (pH 7.5), 2 mM ATP, 10 mM $MgCl_2$, [^{3}H]-thymidine (~1 µM, 0.05–0.2 µCi per reaction), and enzyme extract equivalent to 1×10^6 cells. After incubation at 37 °C for 5 min, reactions were terminated by addition of 0.5 M EDTA. The resulting [^{3}H]-dTMP was captured on DEAE (DE81) filters, which were washed extensively with 50 mM ammonium acetate to remove unreacted substrate. Filters were dried, transferred to scintillation vials, and radioactivity was measured by liquid scintillation counting. TK activity was calculated as nmol/min/10^8 cells, corrected for reaction time and cell equivalents. Negative controls lacking ATP or using heat-inactivated extracts were included to account for nonspecific binding and background radioactivity. [5]

1. White JC, Goldman ID (1981) Methotrexate resistance in an L1210 cell line resulting from increased dihydrofolate reductase, decreased thymidylate synthetase activity, and normal membrane transport. J Biol Chem 256:5722–5727
2. Hisata T (1975) An accurate method for estimating 5-phosphoribosyl 1-pyrophosphate in animal tissues with the use of acid extraction. Anal Biochem 68:448–457. https://doi.org/10.1016/0003-2697(75)90640-5
3. Seegmiller JE, Rosenbloom FM, Kelley WN (1967) Enzyme Defect Associated with a Sex-Linked Human Neurological Disorder and Excessive Purine Synthesis. Science (80-) 155:1682–1684. https://doi.org/10.1126/science.155.3770.1682
4. Kelley WN, Rosenbloom FM, Henderson JF, Seegmiller JE (1967) A specific enzyme defect in gout associated with overproduction of uric acid. Proc Natl Acad Sci U S A 57:1735–9. https://doi.org/10.1073/pnas.57.6.1735
5. van den Berg KJ (1986) Direct assay of thymidine kinase bound to ion-exchange paper for dot spotting and enzyme blotting analysis. Anal Biochem 155:149–154. https://doi.org/10.1016/0003-2697(86)90240-X
